# Fine-Structural Morphology of the Mouthparts of the Polyphagous Invasive Planthopper, *Ricania speculum* (Walker) (Hemiptera: Fulgoromorpha: Ricaniidae)

**DOI:** 10.3390/insects13090843

**Published:** 2022-09-16

**Authors:** Tiantian Gao, Jolanta Brożek, Wu Dai

**Affiliations:** 1 Key Laboratory of Plant Protection Resources and Pest Integrated Management of the Ministry of Education, College of Plant Protection, Northwest A&F University, Yangling, Xianyang 712100, China; 2 Faculty of Natural Sciences, Institute of Biology, Biotechnology and Environmental Protection, University of Silesia, Bankowa 9, 40-007 Katowice, Poland

**Keywords:** Auchenorrhyncha, stylet fascicle, sensilla, scanning electron microscopy

## Abstract

**Simple Summary:**

Mouthparts are the crucial organs for food detection and feeding. Here, the mouthparts of a representative of the planthopper family Ricaniidae are studied and illustrated in detail for the first time and compared to those of other species of plant-feeding Hemiptera.

**Abstract:**

Mouthparts are the crucial sensory and feeding organs associated with food detection and feeding in insects. The Asian ricaniid planthopper *Ricania speculum* (Walker), recently introduced into Europe, can cause severe economic damage by sucking the phloem sap of tea, camphor, citrus, black locust and other plants using piercing-sucking mouthparts. To facilitate comprehensive understanding of feeding mechanisms in the Ricaniidae, the fine structure of the mouthparts of *Ricania speculum* was observed by scanning electron microscopy for the first time. The mouthparts are tubular, consist of a cone-shaped labrum, with a wrinkled epidermis and without sensilla; the tubular labium is divided into three segments: a slender stylet fascicle consisting of two mandibular stylets with four ridged processes and a row of longitudinal striations on the distal part of the outer surface; and two maxillary stylets with a smooth and sharp distal part, interlocked to form a larger food canal and a smaller salivary canal. On the labium, 15 kinds of sensilla of different functions were recognized. Two rows of short sensilla basiconica (SB I) are symmetrically distributed along the labial groove on the first segment. Two pairs of long sensilla basiconica (SB II) (proprioceptors) are on both sides of the labial groove at the junction of the second and third segments. A placoid, flattened sensillum (SPF) is symmetrically located laterally on the proximal end of the last segment and several flattened sensilla campaniformia (SFC) were visible on the ventral side on the second and third segments. The distribution of four types (I–IV) of sensilla cheatica of different lengths on the dorsal surface of the labium is significantly denser than on the lateral and ventral surfaces. The labial apex is divided into dorsal and ventral sensory fields, mainly including uniporous long peg sensilla (I), as well as smaller peg sensilla (II) and nonporous peg sensilla (PGSN) on each dorsal field. These nonporous sensilla basiconica (BSN I and III) occur on the ventral sensory fields and are constant in number and distribution. The nonporous sensilla basiconica (BSN II) are symmetrically arranged near the opening of the stylet fascicle similarly to two oval multiporous plate sensilla (OPSM). The sensilla arrangement is slightly different from that observed in previously studied Fulgoromorpha using scanning electron micrographs, which may reflect differences in feeding preference or behavior.

## 1. Introduction

As phytophagous insects adapt to habitat and host plant changes, such changes may be reflected in changes to the fine structure of their mouthparts [1]. Study of fine-structural differences among species may, therefore, allow inferences regarding feeding habits, the feeding mode and the type of food digested [1,2,3,4,5]. Characteristics of the mouthparts have great value in morphological taxonomy, phylogenetics, feeding ecology, physiology and evolution [6,7,8,9]. In particular, the type, quantity and distributions of sensilla may reflect the function and sensitivity of the mouthparts [10,11].

Prior studies on the mouthparts of Hemiptera mainly focused on their overall morphology, the distribution of sensilla on the labium, and feeding behavior. One study systematically studied the adaptability of the structure and function of the mouthparts and sensilla of Hemiptera in relation to food selection, but mainly concentrated on different groups of Heteroptera [12]. Previous studies of non-heteropteran Hemiptera revealed extensive variation among species and higher groups, with some authors noting that differences in mouthpart structures such as teeth on the mandibular stylets and types and arrangement of labial sensilla may reflect ecological, behavioral and functional differences [13,14,15,16,17,18,19,20,21]. Previous studies on the fine structure of the mouthparts of Fulgoromorpha mainly described the overall morphology of the mouthparts [19], sensilla of the labial tip and locking mechanism of the stylets [13,14,20,21,22]. The labial sensilla characters and their distributions were analyzed in several species of different fulgoromorphan families. Foster [20] showed that there were three kinds of sensilla in the labium of the brown planthopper, including 12 pairs of peg sensilla, 2 pairs of pit sensilla, and 1 pair of dome sensilla of different functions. A study showed in two species of Cixiidae the presence of peg-shaped sensilla on the labial apex [23]. More details on the kinds and arrangement of the labial tip sensilla in species representing different fulgoromorphan families were presented [14]. One study reported a special subapical sensillum, while another verified the presence of this sensillum in Delphacidae, Dictyopharidae and Tettigometridae, but did not find it in Fulgoridae [1,24].

In this study, the black planthopper *Ricania speculum* (Walker) of Ricaniidae was selected as the research object. *R. speculum* is an Asian insect that can feed on more than 60 different woody plant species including several crops in tropical and subtropical areas (citrus, cocoa, coffee, cotton, sorghum, oil palm, apple, sugarcane, tea oil, etc.) and has been reported as an alien invasive species in Europe, where it threatens local plant diversity, including important crops [25,26,27,28,29,30]. One of the visible behaviors of this species is their formation of aggregation clumps in young branches where they puncture the host tissue and absorb plant juice, resulting in weak growth of host plants, leaf curvature and chlorosis; leading to branch death [25,26,27,28]. The white cotton flocculent wax discharged by nymphs can cover the surface of branches or leaves, weakening the photosynthesis of plants, and thus may induce growth decline [25,26,27,28]. The mouthparts show the adaptability to exploit different food sources [1,31]. To facilitate future study of feeding mechanisms and modes of feeding damage, therefore, we studied the ultra-morphology of the mouthparts in *R. speculum*, including distribution of sensilla located on the labium, structural characteristics of stylets and the locking mode of maxillary stylets using scanning electron microscopy. Additionally, we analyzed the possibility of their taxonomic and putative functional significance, so as to further understand the feeding mechanism of *R. speculum* and its adaptability to the environment. 

## 2. Materials and Methods

### 2.1. Insect Collecting

Adults of *R. speculum* used for SEM in this study were collected with sweep nets from Yongle Town, Leishan County, Guizhou Province, on 30 July 2016 and Maliwan Town, Jiangkou County, Guizhou Province on 27 July 2019 and preserved in 75% ethanol.

### 2.2. Scanning Electron Microscopy

Randomly selected specimens of *R. speculum* including adult males (*n* = 5) and females (*n* = 5) were immersed in 75% ethanol and the heads were removed with dissecting needles under a stereomicroscope (Olympus SZX10, Tokyo, Japan), then dipped into 75% ethanol before being placed in an ultrasonic cleaner (KQ118, Kunshan, China) and cleaned 3 times, 5–10 s each time. A graded series of ethanol was then used to dehydrate the specimens for 20 min in 80, 90, and 95% ethanol in turn, followed by twice in 100% ethanol, each time for 30 min. Next, the specimens were soaked in a graded series of ethanol: tert-Butanol solution at the volume ratio of 3:1, 1:1 and 1:3 for 15 min at each ratio, then soaked in 100% tert-butanol for 30 min before being placed into a freeze-drier (VFD-21S, SHINKKUVD, Tokyo, Japan) with liquid CO2 for 3 h. The dried samples were dissected under the stereomicroscope before being mounted on aluminum stubs using double-sided copper sticky tape and coated with gold/palladium (40/60) in a high-resolution sputter coater (MSP-1S, SHINKKU VD, Ibaraki, Japan). Specimens were observed and photographed under the scanning electron microscope (Nova Nano SEM-450, FEI, Hillsboro, OR, USA) at 5–10 kV.

### 2.3. Image Processing and Data Analysis

The obtained electron microscope images were processed by Photoshop 2019 (Adobe Systems, San Jose, CA, USA) and used to classify characteristics of each segment of the *R. speculum* labium, the types of sensilla, and the morphology of the stylets. Statistical analyses were performed using SPSS 19.0 (SPSS, Chicago, IL, USA). The differences in the length of the labrum, labium, mandibular stylets and maxillary stylets between the sexes were analyzed using independent-samples *t*-test. Before analyses, the data were tested by Shapiro–Wilk for normality and satisfied the homogeneity of variance assumption in Levene’s test. The length of each segment of the male and female adults was tested by a nonparametric test (the Mann-Whitney U test) to determine whether the difference was significant because the data did not meet the normal distribution. Data were reported as having means of ± S.E. or medians (min–max). The level of significance in all tests was set at 0.05.

### 2.4. Terminology

The terminology used for sensilla follows the nomenclature system of Altner and Prillinger [32] with more specialized nomenclature for the labial apex sensilla following Brożek and Bourgoin [14]. For terms describing the stylet linkage mechanism, we followed Brożek et al. [33].

## 3. Results

### 3.1. Gross Morphology of Mouthparts

**Figure 1:**
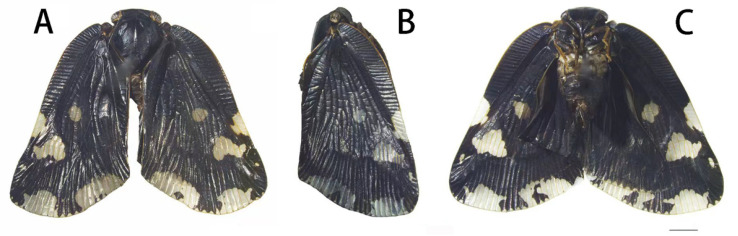
Habitus of adult *Ricania speculum*. (**A**) Dorsal view. (**B**) Lateral view. (**C**) Ventral view. Bar = 2 mm.

**Figure 2:**
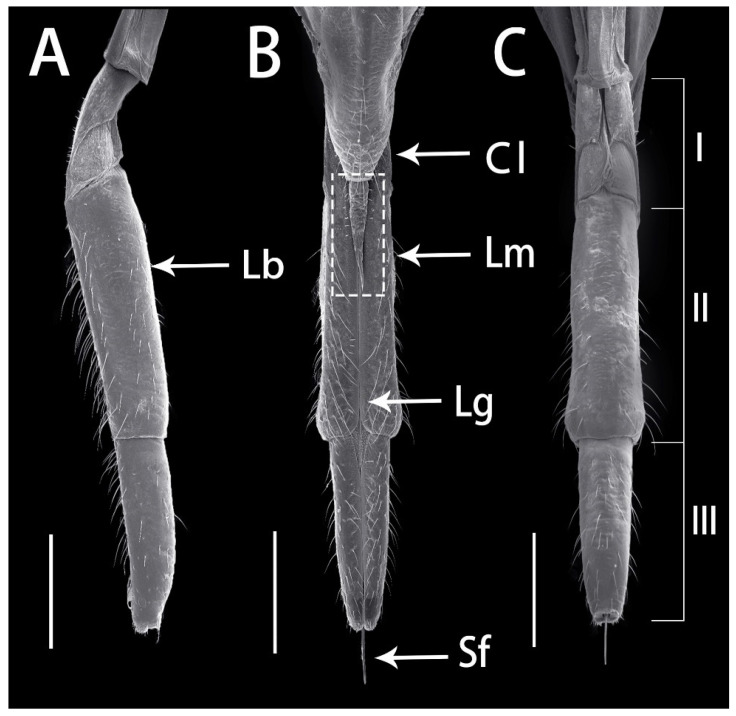
SEM of the mouthparts of adult *Ricania speculum* female. (**A**) Lateral view. (**B**) Dorsal view. (**C**) Ventral view showing the three labial segments. Cl, clypeus; Lm, labrum; Lb, labium; Lg, labial groove; Sf, stylet fascicle; I–III, segments of labium. Bars: (**A**–**C**) = 250 μm.

The mouthparts of *R. speculum* are attached to the posterior part of the head capsule (Figure 1) and composed of a conical labrum (Lm), a tubular labium (Lb) and four stylets in an interlocking fascicle (Sf) (Figure 2A–C). During feeding, the mouthparts rotate from the membranous base and extend downward until becoming perpendicular to the body and the host plant surface, allowing the stylets to penetrate the plant tissue and suck in the plant sap. After feeding, the mouthparts return to their more horizontal pre-feeding position. The labrum surface is wrinkled, with transverse cuticular processes at the base and longitudinal ridges at the end, with no sensilla. The labium is subdivided into three segments of different lengths (Figure 2C), possessing a deep longitudinal labial groove (Lg) on the anterior surface enclosing the stylet fascicle (Sf), which consists of two inner maxillary stylets (Mx) surrounded by two shorter mandibular stylets (Md). The relationship between the length of each segment of the labium is: I < III < II, and the second segment is significantly longer than the other two (first segment, df = 16, *z* = 3.464, *p* = 0.001; third segment, df = 16, *z* = 3.464, *p* = 0.001) (Table 1). No obvious differences were noted in the mouthpart structures of the labium between females and males except for the length of the labrum and labium (in Lm, df = 6, *t* = 3.167, *p* = 0.019; in Lb, df = 8, *t* = 4.259, *p* = 0.002). 

**Figure 3:**
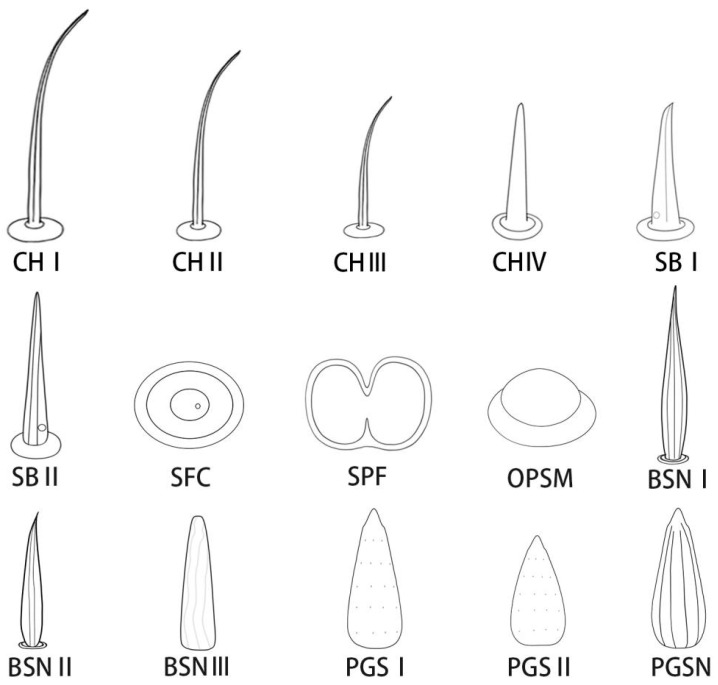
Diagrams of different types of sensilla on mouthparts of adult *R. speculum*. CH I, sensilla chaetica I; CH II, sensilla chaetica II; CH III, sensilla chaetica III; CH IV, sensillum chaeticum IV; SB I, sensilla basiconica I; SB II, sensilla basiconica II; SFC, flattened campaniform sensilla; SPF, lateral placoid-shaped sensilla; OPSM, oval plate sensillum, multiparous; BSN I, sensillum basiconicum, nonporous I; BSN II, sensillum basiconicum, nonporous II; BSN III, sensillum basiconicum, nonporous III; PGS I, peg sensilla I; PGS II, peg sensilla II; PGSN peg sensillum, nonporous.

Different types of sensilla (Figure 3, Table 2) are distributed symmetrically on either side of the labial groove and on the labial apex. The labial apex is divided into two lobes (right and left) and the tip of the stylet fascicle extends from its central opening. Each lobe includes dorsal and ventral sensory fields, with different types of sensilla distributed over their surfaces.

### 3.2. Labrum

**Figure 4:**
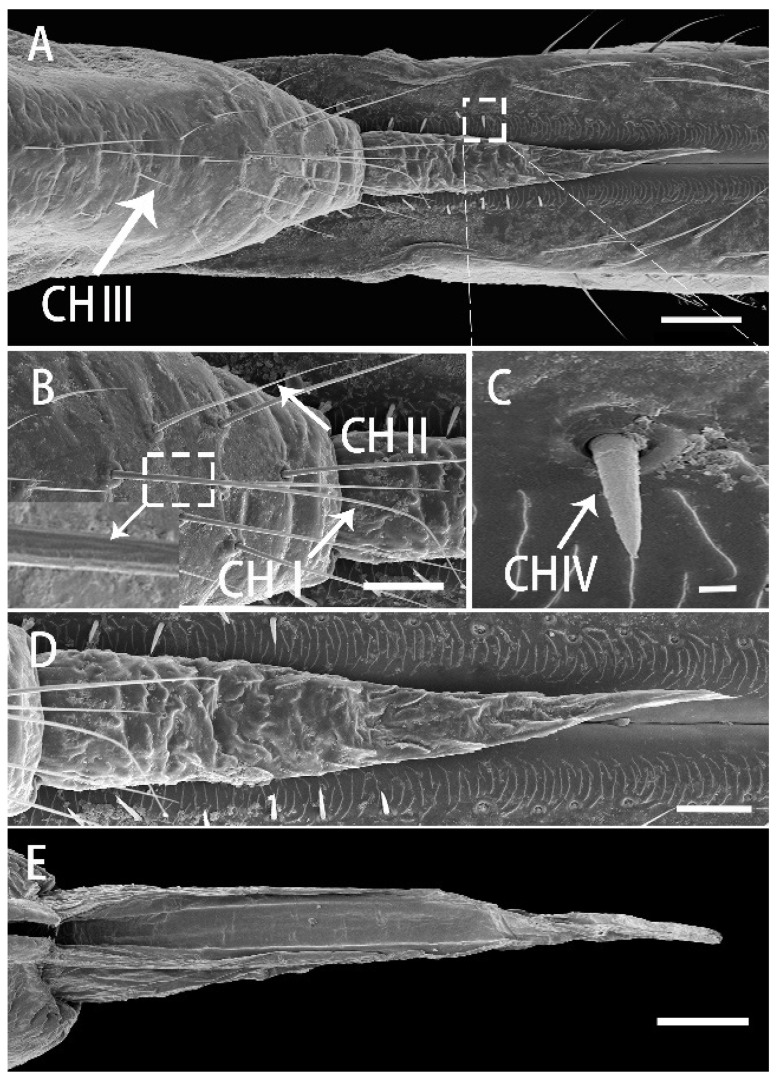
SEM of the labrum of adult *R. speculum*. (**A**) Dorsal view of the labrum. (**B**) Enlarged view of middle part of (**A**) showing posterior clypeus. (**C**) Enlarged view of outlined box of (**A**) showing sensilla chaetica IV (CH IV). (**B**) Enlarged view of middle part of (**A**) showing posterior clypeus. (**D**) Enlarged dorsal view of labrum. (**E**) Enlarged ventral view of labrum. CH I–IV, sensilla chaetica I–IV. (**A**–**D**) from female; (**E**) from male. Bars: (**A**) = 50 μm; (**B**) = 20 μm; (**C**) = 5 μm; (**D**,**E**) = 25 μm.

The conical labrum has a wide base and tapers gradually to the end, connects to the clypeus at the base and overlaps the labial groove up to one-third the length of the dorsal surface of segment II (Figure 4A). A large number of irregular transverse folds are present at the base but dwindle toward the apex, transitioning to irregular longitudinal ridges (Figure 4D). No sensilla are present on the exterior surface of the labrum, but a small number of CH I to CH III (Figure 3) are distributed at the base end of the clypeus (Figure 4A, B). The inner surface of the labrum has several transverse striations and a large longitudinal groove that covers the stylet fascicle (Figure 4E). 

### 3.3. Labium

**Figure 5:**
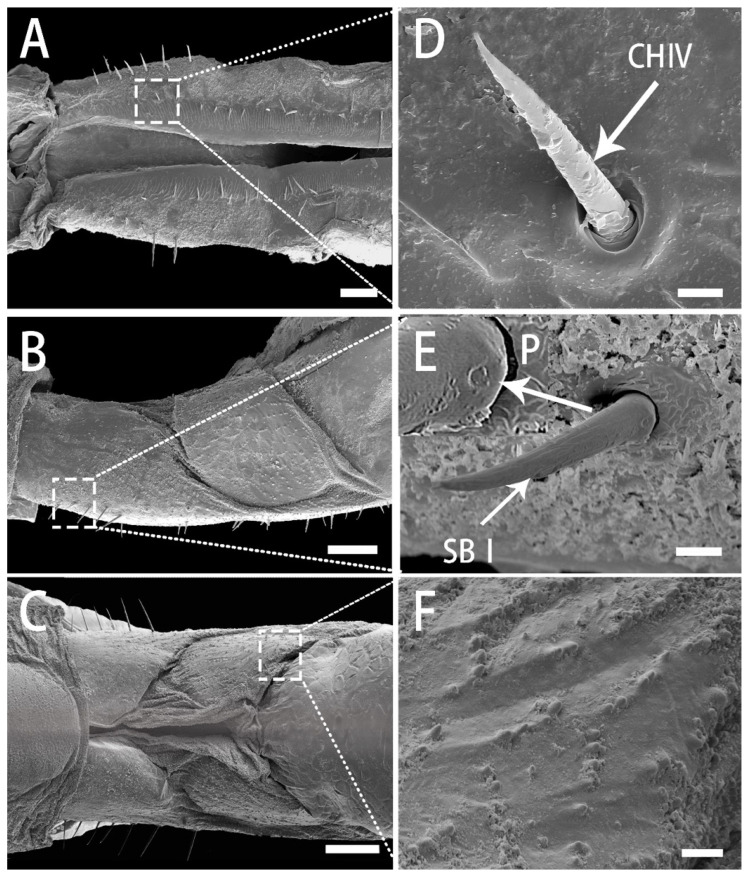
SEM of first segment of labium of adult *R. speculum*. (**A**) Dorsal view. (**B**) Lateral view. (**C**) Ventral view. (**D**) Enlarged view of outlined box of (**A**), showing the sensilla chaetica IV (CH IV) (white arrow). (**E**) Enlarged view of outlined box of (**B**) showing the sensilla basiconica I (SB I) (white arrow) and prominent pore (P) at base of SB I (white arrow). (**F**) Enlarged view of outlined box of (**C**), showing the granular protrusions. (**A**,**B**,**D**,**E**) from female; (**C**,**F**) from male. Bars: (**A**–**C**) = 50 μm; (**D**,**E**) = 2 μm; (**F**) = 5 μm.

The short segment I of the labium has a ventral surface divided into two pairs of lobes separated by membranous grooves that allow the segment to bend at the middle (Figure 5C). The anterior side of the segment is concealed by the clypeus (Figure 4A) and can only be clearly observed when the labium is removed from the head, but the boundary is indistinct (Figure 5A). A small number of SBI are located on both sides of the dorsal surface, each with longitudinal grooves on the surface and a pore on the base (Figure 5B,E). On both sides of the labial groove, a row of irregular ripples are perpendicularly arranged along the length of the groove, and smooth nonporous sensilla chaetica (CH IV), which are directed toward the labium groove (Figure 5D). The smooth ventral surface bears a longitudinal fracture in the center, which is membranous (endocuticular layer) with dense granular protrusions that extend to the sides (Figure 5F). 

**Figure 6:**
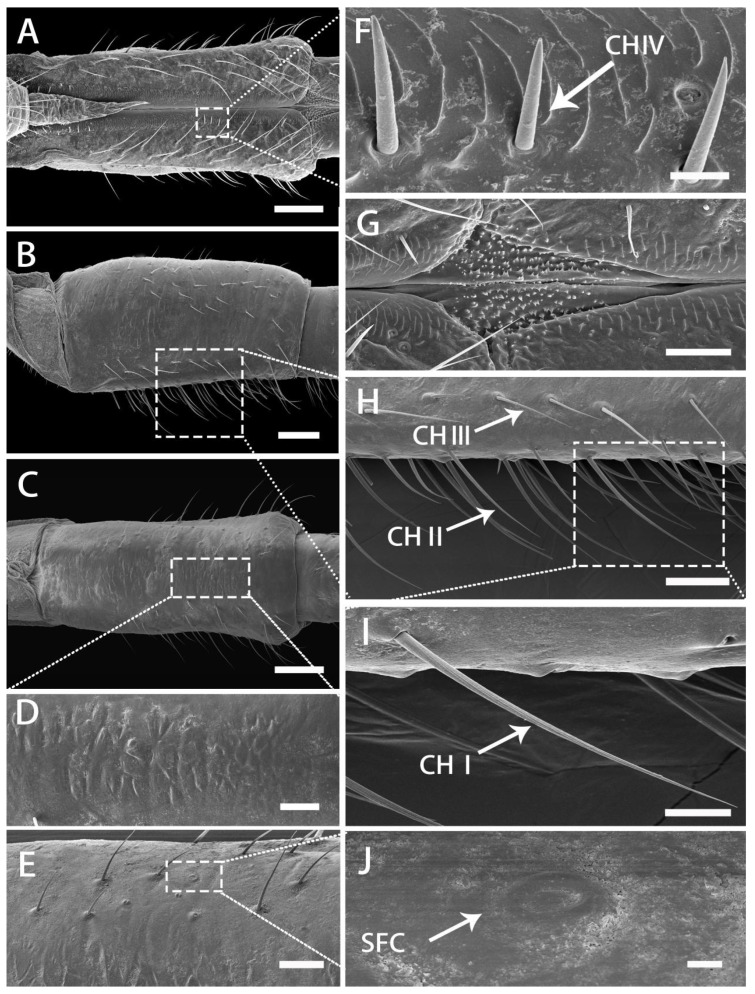
SEM of second segment of labium of adult *R. speculum*. (**A**) Dorsal view. (**B**) Lateral view. (**C**) Ventral view. (**D**) Enlarged view of outlined box of (**C**) showing fish-scale processes. (**E**) Enlarged view of (**C**) showing the upper edge of ventral surface. (**F**) Enlarged view of outlined box of (**A**) showing sensilla chaetica IV (CH IV). (**G**) Enlarged view of outlined box of (**A**) showing spinous processes at the junction of segment II and Ⅲ. (**H**) Enlarged view of (**B**) showing CH I, CH II and CH III at the lower edge of lateral surface. (**I**) Enlarged view of outlined box of (**H**) showing sensilla chaetica I (CH I). (**J**) Enlarged view of outlined box of (**E**) showing flattened campaniform sensilla (SFC). (**A**,**C**–**J**) from male; (**B**,**H**,**I**) from female. Bars: (**A**–**C**) = 100 μm; (**D**) = 20 μm; (**E**) = 25 μm; (**F**) = 5 μm; (**G**,**H**) = 25 μm; (**I**) = 10 μm; (**J**) = 2 μm.

Segment II expands in the middle and at the end slightly, has a fish scale-like surface laterally and ventrally (Figure 6B–D), as well as a rhomboid membranous region covered with microtrichia, which is located at the junction of segments II and Ⅲ (Figure 6G). Some CH II and CH Ⅲ are located on the ventral and lateral surface; however, they are mainly concentrated along the labial groove on the dorsal surface (Figure 6B,C). Near the distal region of the segment, sensilla CH III are less numerous than long sensilla chaetica (CH II). The entire dorsal surface of the segment possesses a wealth of receptors, mainly CH I to CH IV (Figure 6A), with CH I distributed on both sides of the dorsal surface; and is slender with a curved apex and obvious longitudinal grooves (Figure 6A,B,H,I). Some CH II are near the labial groove, whereas CH III appear alternately and are arranged closely with CH I (Figure 6H,I). The distribution of CH IV is similar to that of the first segment, regularly arranged into two rows along the labial groove (Figure 6F). Several SFC are found on the lateral and ventral surfaces but their cuticular depression is indistinct (Figure 6J).

**Figure 7:**
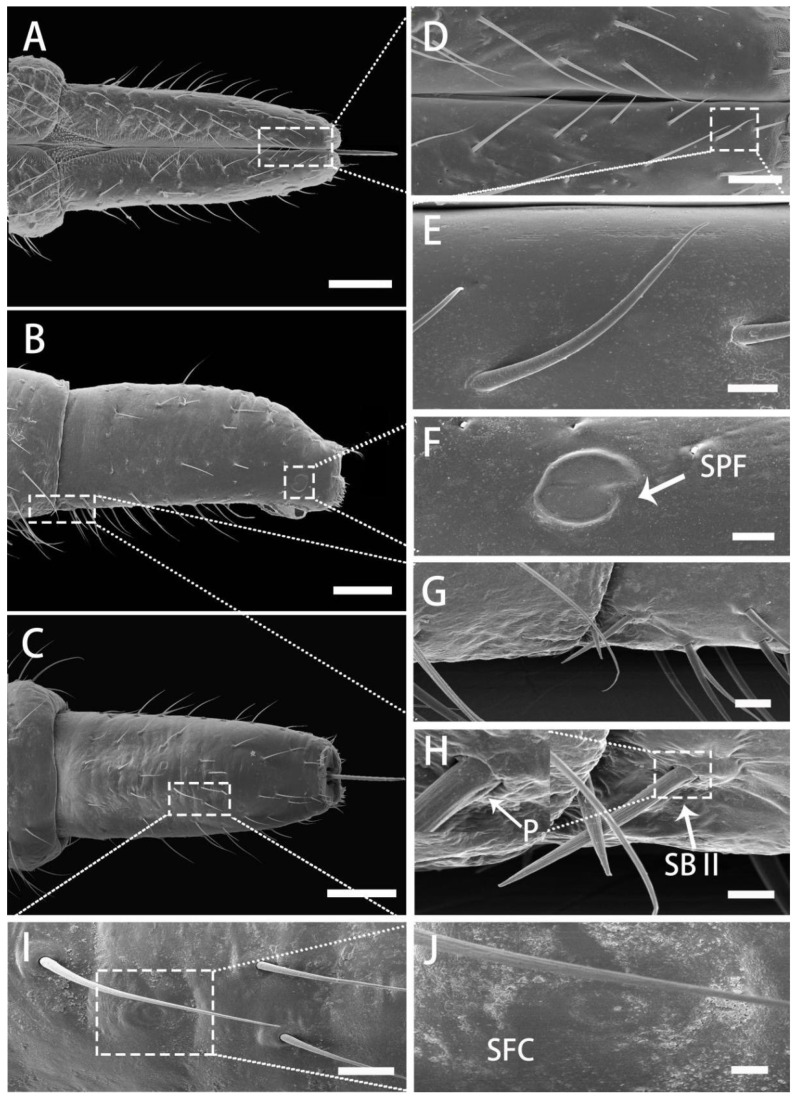
SEM of third segment of labium of adult *R. speculum*. (**A**) Dorsal view. (**B**) Lateral view. **(C**) Ventral view. (**D**) Enlarged view of outlined box of (**A**) showing sensilla at the end of dorsal view of the third segment. (**E**) Enlarged view of outlined box of (**D**) showing sensillum basiconicum, nonporous (BSN II). (**F**) Enlarged view of outlined box of (**B**) showing lateral placoid-shaped sensilla (SPF). (**G**) Enlarged view of outlined box of (**B**) showing sensilla basiconica II (SB II). (**H**) Enlarged view of (**G**) and pore (P) at base of SB II (white arrow). (**I**) Enlarged view of outlined box of (**C**) showing flattened campaniform sensilla (SFC). (**J**) Enlarged view of outlined box of (**I**) showing flattened campaniform sensilla (SFC). (**A**,**C**,**D**,**E**,**I**,**J**) from male; (**B**,**F**,**G**,**H**) from female. Bars: (**A**–**C**) = 100 μm; (**D**) = 20 μm; (**E**) = 5 μm; (**F**,**G**) = 10 μm; (**H**) = 5 μm; (**I**) = 10 μm; (**J**) = 2 μm.

The third segment (III) is wide at the base, gradually narrowing to the end, and ventrally is evidently convex (Figure 7A–C). Sensilla of this segment are the most abundant and densely distributed, with a small number of CH I, a large number of CH II and CH III, four SB II, and two SPF localized laterally near the tip of the segment. Several SFC (campaniform sensilla) are visible on the ventral side. CH II and CH III are numerous and distributed irregularly throughout the surface of segment III (Figure 7A–C). CH IV are mostly lacking on both sides of the labial groove, but several BSN II are distributed in the center of the proximal part of the segment. The surface of these sensilla is smooth and their base is inserted into the depression of the cuticle (Figure 7D,E). Thick, short and straight SB II inserted into a socket are found on both sides of the base of the third section, distributed in pairs, with longitudinal grooves on the surface and a pore at the base. On the lateral side, a small number of CH II and CH III are mainly present (Figure 7B), and SPF are arranged on both sides near the labium tip (Figure 7F). On the ventral side, CH II and CH III are located in a small number (Figure 7C). Just like in segment II, a slight amount of SFC (campaniform sensilla) are disseminated on the ventral side (Figure 7I,J).

**Figure 8:**
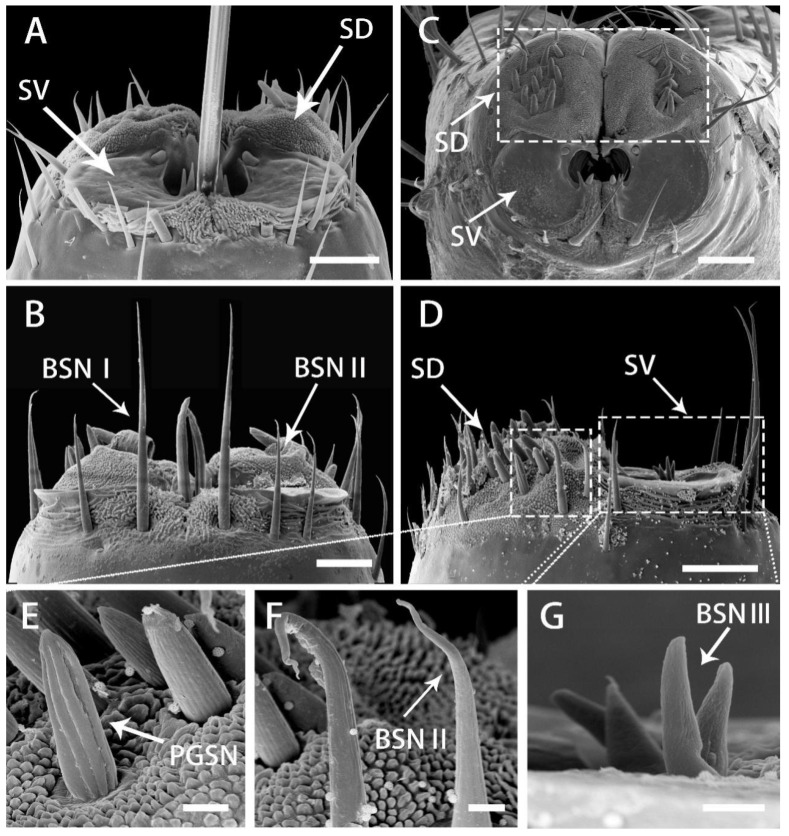
Distribution of various sensilla on tip of labium of adult *R. speculum*. (**A**–**D**) SEM views of labial tip of different individuals showing variation in numbers of sensilla. (**E**,**F**) Enlarged view of the outlined box of (**D**) showing the peg sensillum, nonporous (PGSN) and sensillum basiconicum, nonporous (BSN II) of dorsal sensory field (SD). (**G**) Enlarged view of the ventral sensory field (SV) showing the sensillum basiconicum, nonporous III (BSN III); BSN I, sensillum basiconicum, nonporous, long; BSN II, sensillum basiconicum, nonporous, medium. (**A**,**C**) from male; (**B**–**G**) from female. Bars: (**A**–**D**) = 20 μm; (**E**–**G**) = 2 μm.

**Figure 9:**
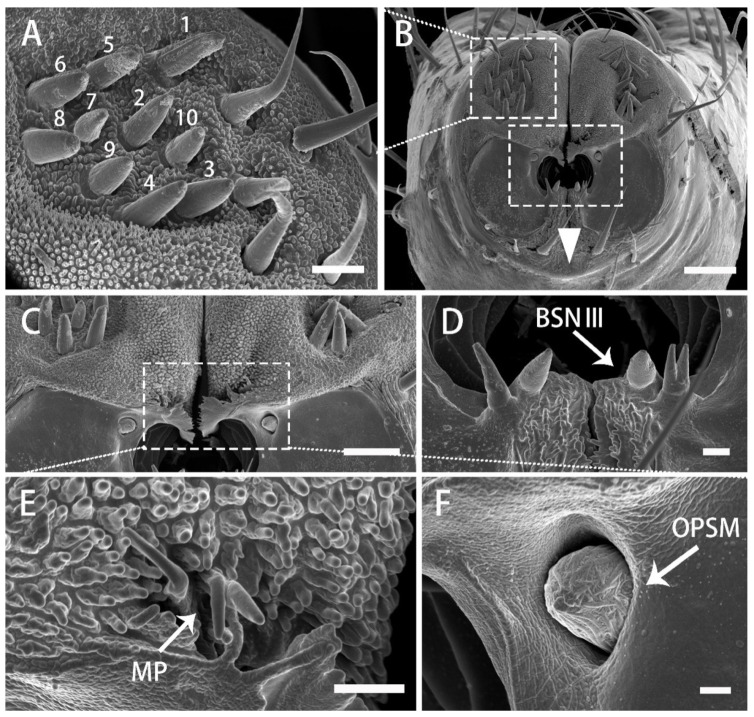
Enlarged views of different types of sensilla and cuticular processes on mouthparts of male *R. speculum*. (**A**) Enlarged view of the outlined box of (**B**) showing the peg sensillum, No. 1; peg sensillum, nonporous (PGSN), Nos. 2–4; peg-like sensilla, long (PGS I), Nos. 5–10; peg-like sensilla, short (PGS II). (**B**) SEM views of labial tip showing various sensilla. (**C**) Enlarged view of the medium of (**B**). (**D**) Enlarged view of the outlined box of (**B**) showing the sensillum basiconicum, nonporous, short (BSN III). (**E**,**F**) Enlarged view of the outlined box of (**C**) showing the cuticular process (MP) and oval plate sensillum, multiporous (OPSM). Bars: (**A**) = 5 μm; (**B**) = 20 μm (**C**) = 10 μm; (**D**,**E**) = 2 μm; (**F**) = 1 μm.

The sensilla at the tip of the labium are the first objects to contact the surface of the host plant. The sensilla are symmetrically and densely distributed on the labial apex. The surface of the concave labial apex is oval, covered with dense papilliform protuberances with lateral folds. The lobes of the labial apex include a pair of dorsal sensory areas (SD) and a ventral sensory area (SV) (Figure 8A–D). The surface of SD is covered with small processes, on which various sensilla are located. The SV is composed of two symmetrical plat plates with smooth surfaces and no protrusions and the sensilla are distributed to the outer edge of the plates. Medially and below the opening from which the stylet fascicle emerges, in the inferior area, two symmetrical protruding folds are covered by cuticular processes. Numerous and specific types of sensilla are comprised in each field (Figure 7A–D). The types and distribution of sensilla are as follows:

Each dorsal sensory field has 1 PGSN, with very obvious longitudinal grooves and without pores (Figure 8E), 3 long PGS I, 6 short PGS II (Figure 8A and Figure 9B), and 13 BSN II, of which 7 short BSN II are closely distributed near the peg sensilla area, 3 long BSN II are located more outside of the peg sensory area and 3 long BSN II are located on both sides of the junction of SD and SV in a triangular distribution (Figure 8F and Figure 9A,B).

On the ventral sensory field, 2 OPSM are symmetrically located on the outer edge of the stylet fascicle hole, near the ventral side (Figure 9C,F). Four particularly short clusters of the BSN III are situated on the inner edge of the stylet fascicle hole, near the dorsal side (Figure 8G and Figure 9D). On the center of the extreme of the dorsal sensory field, 2 long BSN I and 2 BSN II are symmetrically located on both sides (Figure 8B). At the center of the junction of SD and SV, close to both sides of the labial groove, are two symmetrical areas of cuticular processes (Figure 9E).

### 3.4. Stylet Fascicle

**Figure 10:**
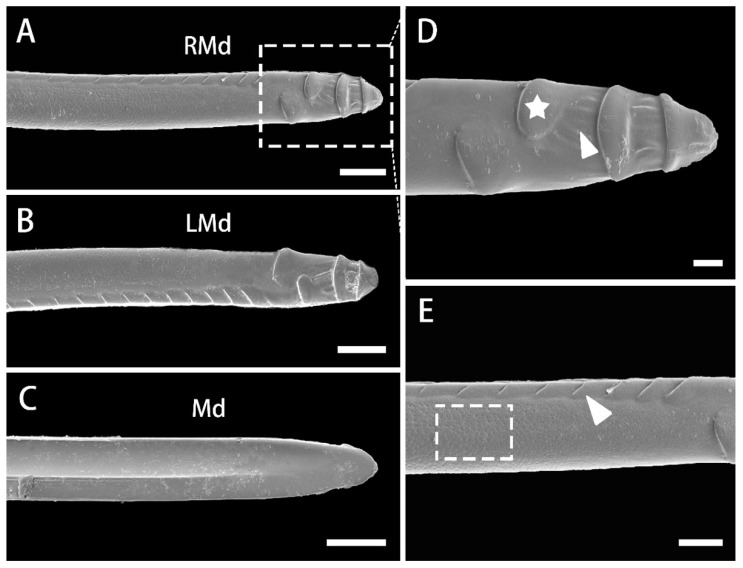
SEM of the mandibular stylets of adult *R. speculum*. (**A**) Tip of external surface of right mandibular stylet (RMd). (**B**) Tip of external surface of left mandibular stylet (LMd). (**C**) Smooth hollow inner surface of mandibular stylet (Md). (**D**) Enlarged view of the outlined box of (**A**) showing oval plate-like prominences (white pentastar) and longitudinal striations (white triangle) on convex external surface. (**E**) Enlarged view of (**A**) showing oblique ribbing of lateral edge of mandibular stylet (Md) (white triangle) and punctuated microsculpture of the whole external surface (white box). (**A**,**D**) from female; (**B**,**C**,**E**) from male. Bars: (**A**–**C**) = 20 μm; (**D**) = 5 μm; (**E**) = 10 μm.

**Figure 11:**
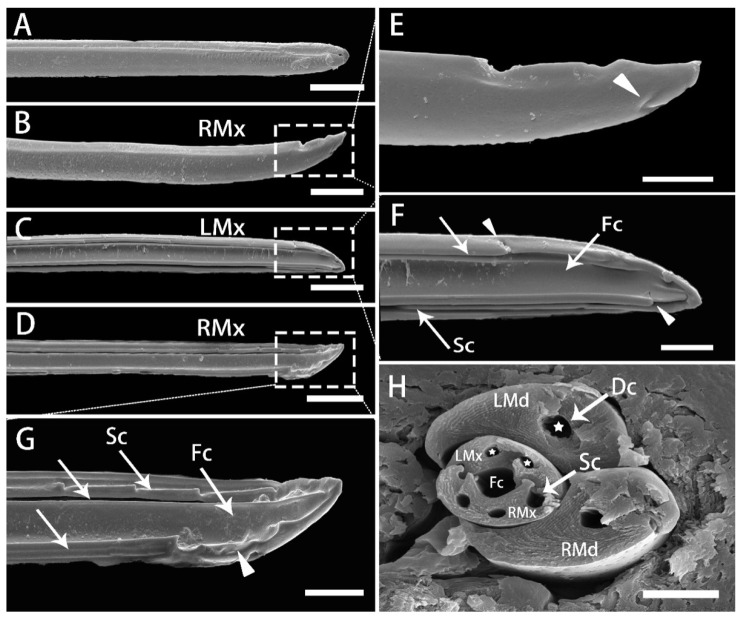
SEM of maxillary stylets of adult *R. speculum*. (**A**) Apex of interlocked maxillary stylets showing outer surface of right maxillary stylet (RMx). (**B**) Smooth external surface of right maxillary stylet (RMx). (**C**) Inner surface of left maxillary stylet (LMx). (**D**) Inner surface of right maxillary stylet (RMx). (**E**) Enlarged view of the outlined box of (**B**) showing the apex of external surface and a “V” depression at the edge of the end of the right maxillary stylet (RMx) (white triangle). (**F**) Enlarged view of the outlined box of (**C**) inner surface of left maxillary stylet (LMx) showing the food canal (Fc), salivary canal (Sc), an interlocking edge (white arrow) and two slit-like openings (white triangle). (**G**) Enlarged view of the outlined box of (**D**) inner surface of the right maxillary stylet (RMx) showing the food canal (Fc), salivary canal (Sc), two interlocking edges (white arrow) and a large gap (white triangle). (**H**) Cross section of the stylet fascicle through the fourth labial segment, showing the shape of two mandibular stylets (Md), two maxillary stylets (Mx), food canal (Fc), salivary canal (Sc) (white arrow) and dendritic canals (Dc) (white penta-star). (**A**,**D**,**G**,**H**) from female; (**B**,**C**,**E**,**F**) from male. Bars: (**A**–**D**) = 20 μm; (**E**–**H**) = 5 μm.

The stylet fascicle consists of pairs of mandibular stylets located on both sides of the interlocking pair of maxillary stylets, the whole located in the labial groove. The maxillary stylets are significantly longer than the mandibular stylets (df = 14, t = 5.333, *p* = 0.000) and protrude from the labium tip (Figure 10A–C and Figure 11A–D). Four irregular transverse prominences are situated on the terminal external surface of the mandibular stylet (Figure 10D). Between these prominences are longitudinal striations. The external oval plate-like prominences help the insect to fix the mouthparts in plant tissue during feeding and support insertion of the maxillary stylets. The lateral edge of the mandibular stylets bears a row of oblique ribbing decreasing from the mandibular tip to the basal part (Figure 10E). The microsculpture of the external surface of the whole stylet is finely punctate (Figure 10E). The inner side of the mandibular stylet is smooth and concave, enclosing the maxillary stylets. The dorsal external ridge of the mandibles is also smooth (Figure 10C). The length of the mandibular and maxillary stylets was significantly different between the sexes (in Md, df = 6, t = 4.527, *p* = 0.002; in Mx, df = 6, t = 4.072, *p* = 0.007).

In contrast to the mandibles, the maxillary stylets are long, asymmetrical and complex. They are smooth on the external surface, and interlock with each other through the internal longitudinal grooves along their length (Figure 11A,B). Distally, an obvious “V” depression is present at the edge (Figure 11E). The inner surface has a wide food channel (Fc) and a smaller salivary channel (Sc). However, the morphology of the inner side of the LMx and RMx is slightly different. The LMx has a complementary and inversely matched shape to the edges to interlock with two edges on the right maxilla, and possesses part of the food channel and a full-shaped, thin and deep salivary channel, with two oblique openings on both sides of the end. On the right one, one part of the food channel is located, similar to that of the left maxilla, but the salivary channel part is shallower than in the left maxilla. In addition, the outer edge near the end of the food channel is shown with a large gap with an uneven edge (Figure 11F,G). Near the end of the left maxillary stylet, a small oblique gap exists at the end of the food and salivary channels, (Figure 11F). In addition, another oblique gap exists at the proximal end of the interlocking canal of the left maxillary stylets, which is speculated to be related to the linkage mechanism of the maxillary stylets (Figure 11F); the same applies to the right maxillary stylet (Figure 11G).

**Figure 12:**
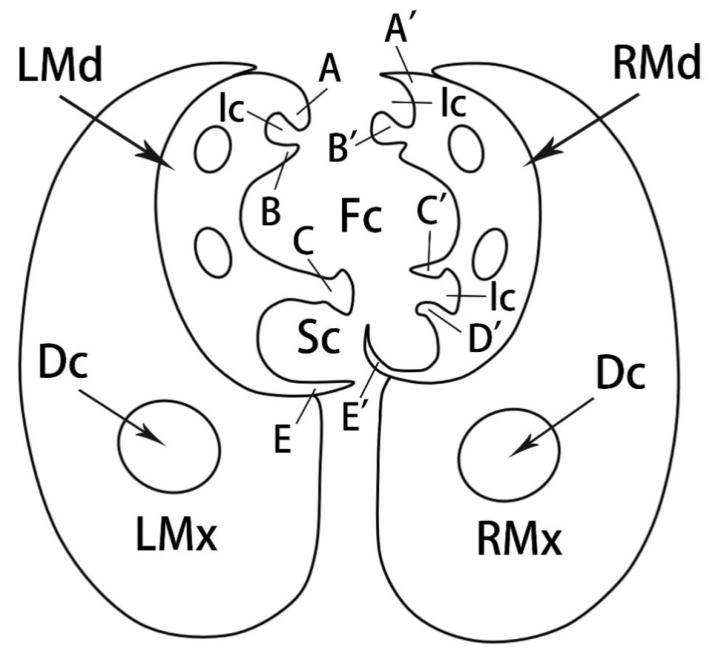
Diagram of cross-section of stylet fascicle through the third labial segment of adult *R. speculum*. LMd, left mandibular stylet; RMd, right mandibular stylet; LMx, left maxillary stylet; RMx, right maxillary stylet; Fc, food canal; Sc, salivary canal; Ic, interlocking canal; Dc, dendritic canals; A, hooked upper left process of dorsal lock; A’, straight upper right process of dorsal lock; B, straight lower left process of dorsal lock; B’, hooked lower right process of dorsal lock; C, T-shaped left process of middle lock; C’, hooked upper right process of middle lock; D’, hooked lower right process of middle lock; E, straight lower left process of ventral lock; E’, hooked lower right process of ventral lock.

The cross-section of the stylet fascicle shows that each mandibular stylet has a dendritic canal and each maxillary stylet has two smaller dendritic canals that run throughout the stylet centrally. In cross-section, the mandibular stylets are approximately semicircular; the maxillary stylets are crescent-shaped and are interlocked by a connecting system composed of T-shaped, hooked, and straight processes (Figure 11H and Figure 12). The food canal is located in the center due to the symmetrically concave inner walls of the two maxillary stylets together forming an ellipse; the salivary canal is located laterally on the inner side of the right stylet, and the interlocking maxillae form a near circle.

## 4. Discussion

Hemiptera insects have highly developed piercing-sucking mouthparts that play an important role in host detection, tissue puncture and pathogen transmission [13]. Comparable to the diversity of host plants and microhabitats used by these insects, their mouthparts also exhibit a high diversity of fine-structural modifications reflecting adaptation [9,18]. Previous research on the mouthpart morphology of Hemiptera has only begun to show the extent of this morphological diversity and only a few studies have so far described and illustrated the fine structural details of the mouthparts of Fulgoromorpha [34,35,36,37].

Here, we provide the first detailed description and illustrations of the mouthparts of a ricaniid planthopper, *R. speculum*. Our observations indicate that the mouthpart structure of *R. speculum* is similar to that of other Auchenorrhyncha in overall structure [19,21,34,35,38,39,40,41], and also shares several basic characteristics with mouthparts of other Hemiptera (e.g., Sternorrhyncha) [16,42,43]. Nevertheless, we note many important differences, particularly in fine structural details, that may be useful for taxonomy and correlate with particular feeding adaptations.

### 4.1. Labrum Structure

Although the general structure of the labrum of hemipteran insects is conservative, the formation, density and distribution of the triangular spines on the surface differ among the species studied. The labrum of Cicadoidea is long and shovel-shaped, with many sensilla trichodea and a squamous structure on the basal surface. The labrum of Cercopidae is also shovel-shaped but mostly smooth and with no sensilla, and only dense squamous processes are present. The cuticular processes at the base of the labrum of Membracidae are petal-shaped, and palm-shaped on both sides and the end. Spinous cuticular processes cover all the labrum of the Cicadellidae and sensilla coeloconica are present. The triangular conical labrum of Fulgoromorpha has folds on the surface and sensilla are present in some groups [44,45,46]. *Lycorma delicatula* has transverse folds on the labrum surface, with few CH2 randomly arranged [36,45]. The labrum of *Diostrombus politus* has folds on the base but is smooth on the end, with no sensilla [37]. The rhombic has small protrusions on the labrum of the *Dictyophara sinica* whereas the labrum of *Sogatella furcifera* is covered with small processes and a few sensilla trichodea [34,47,48]. The shape of the labrum of *R. speculum* is similar to that of other Fulgoromorpha, all of which are triangular cones. The labrum base is wider than the apex; surface folds are stacked transversely at the base but longitudinal ridges are present toward the end and the whole surface lacks sensilla.

### 4.2. Labium Shape, and Arrangement Sensilla on the Labial Surface

The labium of Hemiptera may be divided into one-to-five segments; however, most species have either three or four segments [49]. In Fulgoromorpha, the labium is usually three-segmented, but *Dictyophara sinica* possess four segments, the same number as in the apple aphid and the citrus aphid [16,42,43]. Other fulgoromorphan species, *Lycorma delicatula* and *Greeni deaficicola* have been reported to have five labial segments [49]. The second segment of the labium is longest in most Fulgoromorpha, but the length of each segment is similar in Delphacidae [17,19,34,41,45,46,48]. Like in most Fulgoromorpha, the labium of *R. speculum* is long, tubular and divided into three segments. The first segment is relatively short while the other two segments are much longer, and the second is significantly longer than the other segments. The demarcation of segments one and two on the dorsal side is not obvious but from the ventral side, the first segment (four sclerites) is evidently divided by a membranous area.

The types and distribution of sensilla are significantly different among different Hemiptera. Therefore, some labial sensilla can be used as an important basis for taxonomy. The labium of Fulgoromorpha is usually equipped with different sets of sensilla chaetica, basiconica, placodea and coeloconica. Differences in the abundance, morphology and arrangement of these sensilla were reported among the species studied [14]. The labium of some Heteroptera has flat-concave flattened nonporous sensilla (campaniform sensilla-mechanoreceptors) similar to those of some species of Fulgoromorpha [14,36]. In *R. speculum*, these sensilla were observed on the ventral side of the last labial segment. Two pairs of campaniform sensilla located at the end of the second segment of the labium are also found in Pentatomidae and Reduviidae, which proved to function as proprioceptors [44,50]. These sensilla may respond to the pressure caused by the movement of the labium [51], and the holes on the sensilla surface may be the external manifestations of insect-molting channels [52]. Nevertheless, the campaniform sensilla belong to a conservative sensory group because they are present in most insects in almost the same shape.

In most taxa, the other types of the proprioceptor are usually identified as sensilla basiconica and located at the junction between the labial segments (e.g., of the fourth and fifth segment in aphids) [9], and are responsible for sensing the bending degree of the segments [46]. The two pairs of sensilla basiconica at the joint of the fourth and fifth segment were found in *Lycorma delicatula* (Fulgoridae) [36]. In *R. speculum*, the sensillum basiconicum between the last two segments is the proprioceptor sensing the degree of bending between the segments. 

In Fulgoromorpha, the sensilla located laterally on the subapical region of the labium differ in form [14]; however, some of them are classified as sensilla coeloconica. A pair of sensilla coeloconica is laterally and symmetrically distributed at the end of the labium in *Sogatella furcifera* [47], while other Fulgoromorpha species (e.g., *Lycorma delicatula*) have a pair of oval concave sensilla placodea [36]. *R. speculum* shows placoid sensilla (SPF) similar to *Pochazia fuscata* (Ricaniidae) and some species of the remaining families (Fulgoridae, Achilidae Dictyopharidae, Tropiduchidae) [14]. Nevertheless, one study observed that subapical lateral sensilla in *Diostrombus politus* and *Proutista moesta* (Derbidae) have bifurcate elevated cone-like sensilla and elevated cone-like sensilla embedded in shallow concavities [37]. 

Sensilla coelconica are reported in many hemipteran species from different parts of the body. They are distributed on the labium of most groups of Cicadomorpha but are lacking in several species of Cicadellidae and Aphrophoridae. In addition, the sensilla coeloconica shows different subtypes depending on their function. They are embedded in a shallow cuticlular cavity as a short cone (peg), but in Deltocephalinae, they are petal-shaped. Non-porous sensilla coeloconica may be temperature and humidity sensors while those with pores have olfactory functions that may sense chemical stimuli to locate hosts or identify pheromones [53,54,55].

The most abundant and common types of sensilla on the labium are sensilla chaetica, and their different distribution and size may be analyzed to find the characteristic patterns. A study described three subtypes (different lengths) of sensilla chaetica on the labium in two species of Derbidae and pointed out a difference among species in the number of sensilla [37]. According to this study, the most abundant were CH1 and CH2, but the longest CH3 are singular and located near the apex of the labium. The labium of *R. speculum* is densely covered by sensilla chaetica in four morphological forms (CH I–CH IV), which possibly have a mechanical function; the longest sensillum chaeticum on the proximal end probably identifies feeding sites by touching plant surfaces. 

### 4.3. Labial Tip Sensilla

As the first structures to contact the host, labial tip receptors play a crucial role in host recognition. As one study noted, one sensory area is present on the labial apex of Coleorrhyncha, two in Cicadomorpha and three in Fulgoromorpha [33]. Moreover, significant differences exist in the labial apex sensilla among different groups of Heteroptera, with one sensory area having 11 or 12 pairs of sensilla in Pentatomidae and Pyrrhocoridae and 11 pairs in Coreidae and Lygaeidae, but is more variable in Reduviidae [56]. In some studies of Miridae, a similar number of uniporous gustatory sensilla are placed more dorsally, and a few aporous mechanoreceptors are located ventrally on both areas of the labium tip (11–12). Generally, within a particular species, the patterns of sensilla arrangement are stable [48,57,58]. 

A study of the labial apex structures of 15 species of Fulgoromorpha noted differences in the shape, number and arrangement of sensilla with different functions [14]. Generally, the uniporous peg or basiconic sensilla were classified as gustatory sensilla and singular multiporous peg sensilla were described as olfactory sensilla. Singular nonporous coeloconic sensilla were indicted as the thermo-hygroreceptors. The other nonporous sensilla types were usually categorized as different mechanosensilla. They recognized some additional, specialized types of sensilla with different functions including dome-like (may be chemical receptors), clavate (likely to be gustatory) and bristle-like (may be mechanical or chemical receptors) sensilla. Other studies provided additional data on the presence of such sensilla in additional species. For example, the dorsal sensory region of *Sogatella furcifera* bears 10 pairs of sensilla, including 1 dome sensilla and 9 peg sensilla, and the ventral sensory field has 2 pairs of sensilla basiconica. One study found six kinds of sensilla on the dorsal sensory fields of the labial apex of *Lycorma delicatula*, with forficate sensilla, finger-like sensilla and clavate sensilla rarely seen in other Fulgoromorphan insects [36].

The labial tip sensilla were previously analyzed in another species of Ricaniidae, *Pochazia fuscata* [14]. Our study indicates that the sensilla of *R. speculum* are similar. The same basic types and quality of the peg gustatory sensilla are present in both species. Nevertheless, these species differ, because only *R. speculum* has two OPSM multiporous sensilla (probably with the olfactory function) symmetrically located on the outer edge of the stylet fascicle, near the ventral side of the dorsal sensory field; R. *speculum* also has one additional type of sensilla basiconica that is not present on *P. fuscata*.

Different types of sensilla at the labial apex cooperate with each other in the feeding process. They may identify the host and feel the relative positions of the stylets in the host plant [20,41,46,59,60,61]. The shape and position of sensilla reflect functional differentiation in feeding behavior to some extent, such as whether there are pores on the surface of the sensilla, or the sensilla is located on the surface of the labrum, labium, internode or labial apex [32,40,62].

The present study determined differences in sensilla morphology and function in *R. speculum* compared with those of previously studied fulgoromorphan species. Additional comparative studies on other fulgoromorphan species are needed to reveal larger-scale patterns of variation that may reflect feeding adaptations and phylogenetic relationships. At present, the definitions of some sensilla types are not perfect, and need clarification through detailed ultrastructural studies based on transmission electron microscopy.

### 4.4. Fine Morphology of the Stylets

Few prior studies of hemipteran mouthparts have attempted to reveal patterns of morphological differentiation that may be associated with different feeding strategies. In particular, some differences in the shape and dentition of the mandibular and maxillary stylets have been associated with predation versus herbivory (sap or seed feeding) [12,13,44,56,63]. 

Within the Heteroptera, apart from the differently shaped external spines, teeth or plates of the mandibles [12,63], the inner squamous structures have been observed in some species of phytophagous bugs including Largidae [64], Tingidae [65] and Pentatomidae [66], but not in predatory bugs such as Reduviidae [53]. The function of the squamous structures is still unclear, but they may be present in most sap-feeding taxa. However, it is supposed that the orientation of the texture is such that the forward thrust of one mandible will cause considerable friction against the outer surface of the adjacent maxillary stylet contributing to its inward deviation [12]. 

In Sternorrhyncha and Auchenorrhyncha species of sap-feeders (mesophyll cells feeding, phloem and xylem feeding), the proximal lateral portion of the mandibular stylets have different numbers of tapered ridges and/or small serrations [18,20,32,36,42,43,44]. For example, some species of Aphidiidae have from 3 to 20, a smaller number are present in Psyllidae and Delphacidae, whereas Cicadellidae and Membracidae have more than 10, and Membracidae and Cicadidae have 4–7 ridges with prominent and sparse teeth on both sides [17,19,34,39,45,46,47]. 

Research so far on Fulgoromorpha has shown that the outer side of the mandibular stylets has 3–6 cuticular ridges [67]. In *Tagosodes orizicolus* (Delphacidae), similarly to *Nilaparvata lugens*, a study identified 5–6 stout protrusions of the mandibular stylets that are only shallowly inserted into the leaf epidermis, while the maxillary stylets extend more deeply into the plant [19]. In *Lycorma delicatula* (Fulgoridae), three oval plate-like prominences of different sizes are present on the terminal part of mandibular stylets and longitudinal striations occur along both sides of the tip [36]. The external surface of mandibular stylets of *R. speculum* has four flat ridge-like protrusions, and rows of longitudinal striations. These structures are very similar to those of *L. delicatula*. The small gap at the end of the food and salivary channels of this species may help saliva flow into the food channel. The oblique gap located at the proximal end of the interlocking canal may be related to the interlocking mechanism of the maxillary stylets. The ends of maxillary stylets are sharp, and the internal structures are stable among fulgoromorphan species, in contrast to the variable external structures of the mandibular stylets.

In sap-feeding Sternorrhyncha and Auchenorrhyncha (aphids, planthoppers and leafhoppers), the outer surfaces and ends of the maxillary stylets are usually smooth [16,67]. The maxillary stylets do not tear the surface tissue of the host but puncture and slide into the appropriate feeding sites within the plant vascular system. This may explain why tips of the maxillae in these groups lack the internal fine teeth present in sap-feeding heteropteran species, which use a “lacerate and flush” feeding strategy [12]. 

The epidermal density of host plants and the penetration depth of stylets may be positively correlated with the number of ridges at the mandibular stylet tips [16,34,46,48,59]. Such ridges are ubiquitous in Hemiptera and help anchor the mouthparts in the epidermis while the maxillary stylets probe more deeply into the plant tissue [16,41,46,48,63]. The longitudinal striations present in some species may assist in anchoring the mouthparts more firmly. Such striations have, so far, only been observed in *R. speculum* and *L. delicatula* [36]. 

The stylet bundles in hemipterans are internally innervated and the nerve canals are frequently visible in the cross-section of the stylets [22]. Usually, one canal is located in each mandible and one canal in the proximal part of each maxilla. In some taxa (Cicadomorpha, Fulgoromorpha), in the distal region of the maxillary stylets, the nerve canal has bifurcated into two branches. In the Coccinea, in each mandible and maxilla, one nerve canal [22] is present but in Cixiidae each maxilla has two canals [14]. A similar situation was observed in membracid and cicadellid species [56] and *L. delicatula* [36]. In *R. speculum*, one nerve canal is present in the mandibular stylet and two in the maxillary stylet, as in other studied Auchenorrhyncha.

## 5. Conclusions

Fine structural features of the mouthparts of the ricaniid planthopper are generally similar to those of previously studied Fulgoromorpha but exhibit several unusual traits. To sum up, the fine structure of the mouth apparatus of *R. speculum* is described in detail from the ultrastructural level for the first time in this study. The shape of the labrum, labium and sensory field of the labial apex, and kinds of sensilla distribution of *R. speculum* are described, as well as the characteristics of stylets and cross section. This study will contribute to a better understanding of the feeding behavior and sensory system of *R. speculum*. Additionally, the novelty of the study introduces a new type of labial tip sensilla found in the *R. speculum* in comparison to *Ricania fuscata*.

## Figures and Tables

**Table 1:** Measurements of the labrum, labium and stylets of adult *Ricania speculum* in both sexes (mean ± SE) obtained from scanning electron microscopy. *n* = sample number. Lb, labium; Lm, labrum; Lb I, the first segment of labium; Lb II, the second segment of labium; Lb III, the third segment of labium; Md, mandibular stylet; Mx, maxillary stylet.

Sex	Position	Length (µm)	Width (µm)	Height (μm)	*n*
Male	Lb	1195.59 ± 24.11			5
	Lm	233.49 ± 5.12	51.74 ± 3.97		4
	Lb I	273.30 ± 10.66	155.64 ± 6.81	104.80 ± 3.43	4
	Lb II	549.78 ± 17.03	186.30 ± 8.19	214.16 ± 12.05	5
	Lb III	387.04 ± 16.07	141.58 ± 5.32	145.87 ± 5.46	5
	Md	1790.51 ± 99.11	8.44 ± 0.43		4
	Mx	2205.33 ± 105.76	5.83 ± 0.26		4
Female	Lb	1250.87 ± 20.73			5
	Lm	247.57 ± 7.27	57.31 ± 4.52		4
	Lb I	281.70 ± 13.51	164.30 ± 8.35	110.30 ± 5.87	4
	Lb II	562.94 ± 11.75	200.04 ± 7.47	240.17 ± 11.10	5
	Lb III	403.87 ± 11.89	153.58 ± 5.26	160.22 ± 4.74	5
	Md	2030.21 ± 64.74	9.98 ± 0.65		4
	Mx	2580.42 ± 150.69	8.45 ± 0.51		4

**Table 2:** Morphometric data for various sensilla of adult *R. speculum* (mean ± SE). *n* = sample size. CH I, sensillum chaeticum, long; CH II, sensillum chaeticum, medium; CH III, sensillum chaeticum, short; CH IV, sensillum chaeticum, shorter; SB I, sensilla basiconica, short; SB II, sensilla basiconica, long; SFC, flattened campaniform sensillum; SPF, lateral placoid-shaped sensilla; OPSM, oval plate sensillum, multiporous; BSN I, sensillum basiconicum, nonporous, long; BSN II, sensillum basiconicum, nonporous, medium; BSN III, sensillum basiconicum, nonporous, short; PGS I, uniporous peg sensillum, long; PGS II, uniporous peg sensillum, short; PGSN peg sensillum, nonporous; Cl, clypeus; Lb, labium; Lg, labial groove; SF-D dorsal sensory field; SF-V ventral sensory field.

Types of Sensilla	Distribution	Length/μm	Width/μm	*n*
CH I	Cl, Lb II-III	104.26 ± 13.52	3.53 ± 0.35	20
CH II	Lb II-III	60.43 ± 9.76	2.68 ± 0.28	20
CH III	Lb I-III	28.87 ± 6.71	1.82 ± 0.21	20
CH IV	Lg	9.85 ± 4.13	1.77 ± 0.44	10
SB I	Lb I	22.69 ± 6.05	2.50 ± 0.41	10
SB II	Lb III	15.84 ± 6.67	2.62 ± 0.31	6
SFC	Lb II-III	6.09 ± 0.72	4.06 ± 0.43	6
SPF	Lb III	24.36 ± 1.73	18.62 ± 0.57	6
OPSM	SF-V	3.92 ± 0.25	3.38 ± 0.42	6
BSN I	SF-V	50.84 ± 3.87	3.24 ± 0.48	8
BSN Ⅱ	SF-V, SF-D	21.75 ± 6.31	2.15 ± 0.44	8
BSN III	SF-V	6.21 ± 1.05	2.08 ± 0.27	6
PGS I	SF-D	8.34 ± 1.335	2.67 ± 0.21	8
PGS II	SF-D	5.35 ± 1.23	2.62 ± 0.27	8
PGSN	SF-D	8.95 ± 1.31	2.78 ± 0.34	6

## Data Availability

The data presented in this study are available in the article.
